# Clinical Evaluation of Violet Light Filtration and High-Resolution Lathing on a Diffractive Extended Depth of Focus IOL

**DOI:** 10.1007/s40123-024-01056-0

**Published:** 2024-10-25

**Authors:** Daniel H. Chang, Andrew A. Kao, Laura K. Huggins, Jacqueline N. Albert, Jacqueline N. Whinery, Brittany M. Camirand

**Affiliations:** Research Department, Empire Eye and Laser Center, Bakersfield, CA USA

**Keywords:** Extended depth of focus IOLs, Dysphotopsia, Low contrast vision, Presbyopia correction, Halos, Starbursts

## Abstract

**Introduction:**

This study is a prospective, randomized, subject/evaluator-masked clinical trial in a single-center clinical setting. The purpose of the study is to compare the clinical performance of Tecnis Symfony Optiblue IOL (models ZXR00V and ZXW150) with violet light filter (VLF) and manufacturing improvements versus Tecnis Symfony IOL (models ZXR00 and ZXT150) with ultraviolet light filter (UVF) in patients undergoing cataract surgery.

**Methods:**

Patients with cataracts aged ≥ 22 years were randomly assigned 1:1 to bilateral implantation with ZXR00V/ZXW150 (VLF group) or ZXR00/ZXT150 (UVF group). Key endpoints at 6 months postoperative included patient reported nighttime dysphotopsia symptoms, 25% low contrast visual acuity with glare, and patient satisfaction.

**Results:**

Sixty patients were implanted with ZXR00V/ZXW150 (30) or ZXR00/ZXT150 (30). At 6 months, the VLF group did not show a statistically significant differences in mean monocular photopic uncorrected distance visual acuity (UCDVA), best-corrected distance visual acuity (BCDVA), uncorrected near visual acuity (UCNVA) (40 cm), or distance-corrected near visual acuity (DCNVA) (40 cm) compared to the UVF group. At 1 month, patients in the VLF group reported significantly less difficulty due to halo (*p* = 0.016) and starburst (*p* = 0.028) symptoms. By the 6 months, dysphotopsia complaints were no longer significantly different between the groups. Although the VLF group trended toward better low contrast visual acuity and patient satisfaction, statistical significance was not reached.

**Conclusions:**

Managing the patients’ expectations is key to achieving success. At the 1-month visit the patients who reported dysphotopsia complaints in the VLF group had significantly less difficulty with starbursts and halos as compared to the UVF group. By the 6-month visit, there was no significant difference between the two groups in the difficulty with starbursts and halos.

**Trial Registration:**

ClinicalTrials.gov identifier, NCT06567834.

## Key Summary Points

**Table:** 

***Why carry out this study?***
Extended depth of focus and multifocal intraocular lenses are associated with nighttime dysphotopsias that can be significant to overall patient satisfaction and functional vision.
Violet light filtration has been shown to reduce nighttime dysphotopsia symptoms in pseudophakic patients.
The purpose of this study is to measure the subjective and objective clinical performance of the Tecnis Symfony Optiblue IOL, with the violet light filtration (VLF) and manufacturing improvements (models ZXR00V and ZXW150), compared with the original Tecnis Symfony IOL, with ultraviolet light filter (UVF, models ZXR00 and ZXT150).
***What was learned from the study?***
Both VLF and UVF groups demonstrated high patient satisfaction with similar distance and near visual acuities, refractive outcomes, low contrast visual acuity, and levels of spectacle independence.
The VLF groups had fewer earlier complaints of dysphotopsias at 1 month. The difference was no longer present at 6 months.
Improvements in testing modalities of low contrast visual acuity, especially with glare, as well as objective testing of dysphotopsia symptoms are needed.

## Introduction

Extended depth of focus (EDOF) intraocular lenses (IOLs) are designed to provide visual quality and visual range, primarily in the distance and intermediate ranges. Like other presbyopia-correcting IOLs, EDOF IOLs can be associated with nighttime dysphotopsia symptoms such as glare, halos, and starbursts, but these are typically less than for multifocal IOLs [1–3]. High energy light filters [4] and more specifically violet light filters (VLF) [5] have been associated with excellent visual quality, high contrast, patient satisfaction, and reduction of dysphotopsia symptoms [6, 7]. Combining violet light filtration with manufacturing improvements reduces stray light and retinal veiling luminance in computer simulations and in vitro models [8]. Designed to mitigate dysphotopsias and enhance contrast vision, the Tecnis Symfony Optiblue IOL (Model ZXR00V, Johnson & Johnson Vision, Irvine, CA) was introduced to replace the Tecnis Symfony IOL (Model ZXR00), which has an ultraviolet light filter (UVF) only. In this study, the clinical performance and patient satisfaction with the Tecnis Symfony Optiblue IOL (models ZXR00V and ZXW150) were compared to that of the Tecnis Symfony IOL (models ZXR00 and ZXT150).

The purpose of this study is to measure the subjective and objective clinical performance and patient satisfaction of the Tecnis Symfony Optiblue IOL (models ZXR00V and ZXW150), with the violet light filtration and manufacturing improvements (VLF group), compared to the original Tecnis Symfony IOL (models ZXR00 and ZXT150) (UVF group).

## Methods

### Study Design

This prospective, bilateral, comparative, masked (patient and study technician), randomized study was conducted at a single site in the USA. This study was approved by the regulatory and institutional review board (IRB), Salus IRB, and was conducted in accordance with the US Code of Federal Regulations, the Declaration of Helsinki, ISO 14155:2011, and all other applicable laws and regulations. All patients provided written informed consent and gave health information access authorization in accordance with Health Insurance Portability and Accountability Act (HIPAA) guidelines prior to participating in the study. This study was initiated in March 2022 and completed in March 2023.

### Inclusion and Exclusion Criteria

Patients were included in the study if they were aged 22 years or older with bilateral cataracts in which bilateral cataract excision with intraocular lens implantation was planned. Each eye had a potential for best-corrected distance visual acuity (BCDVA) of 20/30 Snellen or better, normal corneal topography, corneal astigmatism between 0 and 2.0 diopters, and clear intraocular media other than cataracts. Key exclusion criteria included previous ocular trauma, ocular surgery including corneal refractive surgery, and ocular or systemic disease that could increase risk or confound surgical outcome.

### Randomizations and Procedures

Eligible subjects were randomly assigned 1:1 to bilateral implantation with either Tecnis Symfony Optiblue/Tecnis Symfony Optiblue Toric (ZXR00V/ZXW150, “VLF group”) or Tecnis Symfony/Tecnis Symfony Toric (ZXR00/ZXT150, “UVF group”). Before randomization, surgeons selected the first operated eye on the basis of their standard clinical practice (eye dominance and/or more severe cataracts). All study staff performing vision testing remained masked to the implanted IOL for the duration of the study.

Surgeons used their personalized A-constant and the Barrett Universal II formula. All cases were done with standard small-incision cataract extraction with IOL insertion into the capsular bag. All toric IOLs were positioned along the steep corneal axis to ensure minimum postoperative refractive astigmatism (< 1.0 D). No additional refractive procedures were performed for the duration of the study.

Postoperative manifest refractions were performed using Early Treatment Diabetic Retinopathy Study (ETDRS) chart format on the CTS (Clinical Trials Suite, M&S Technologies, Inc. [Niles, IL]) at 4 m. Monocular and binocular uncorrected distance visual acuity (UCDVA, with + 0.25 D adjustment for optical infinity compensation), monocular best-corrected distance visual acuity (BCDVA, no adjustment to MRx), monocular and binocular uncorrected near visual acuity at 40 cm (UCNVA, no adjustment), and distance-corrected monocular near visual acuity at 40 cm (DCNVA, with − 0.25 D added to ETDRS) were measured under photopic lighting conditions (85 cd/m^2^, 80–110 cd/m^2^ acceptable). Low contrast acuity was tested at 4 m under photopic without glare and mesopic with and without glare conditions using the CTS at the 10% and 25% contrast levels. Binocular uncorrected (+ 0.25 D adjustment) and monocular distance-corrected (no adjustment) low contrast acuity were tested at 1 and 6 months, respectively. Start and end times were recorded at each visit for both technician and investigator.

Visual symptoms, spectacle independence, and patient satisfaction were assessed using the Patient-Reported Visual Symptoms Questionnaire (PRVSQv2), the Patient-Reported Spectacle Independence Questionnaire (PRSIQ), and the Lens Recommendation Questionnaire.

Subjective ocular symptoms were assessed at each postoperative visit by asking “Are you having difficulties with your eyes or vision?” Subject responses were recorded for each eye. Adverse events (AEs) were monitored throughout the study.

### End Points

The primary endpoint was the difference in patient-reported nighttime dysphotopsia symptoms as measured by the PRVSQv2 questionnaire at 6 months. The secondary endpoints were 10% and 25% low contrast visual acuity with glare and patient satisfaction at 6 months. Other endpoints included monocular and binocular uncorrected distance visual acuity (UCDVA), binocular uncorrected near visual acuity (UCNVA) at 40 cm, 10% and 25% low contrast acuity without glare, spectacle use, and time spent with both technician and physician in the examination room.

### Statistical Analysis

Statistical analysis of the data was performed using SAS software Version 9.4. All clinical and performance parameters, as well as all symptoms-related questions, were summarized at months 1 and 6. The results were compared using a parametric* t* test for visual acuity data and the Wilcoxon rank-sum test for questionnaire data. For all subjective symptoms, the subject was required to indicate if the symptom was debilitating. The distribution (number and percentage) of each response by treatment was provided. The percentage of patients reporting debilitation was summarized separately by treatment (VLF group or UVF group) and a test of difference was conducted by the chi-square test.

## Results

Patients underwent bilateral cataract extraction with IOL implantation between March 24 and November 8, 2022. All patients completed 100% of the study visits for both first and second eyes. Patient demographics were similar between the two IOL groups and while there was no significant difference in any demographic, there was a trend toward more older patients in the VLF group (Table 1). One patient from each group was excluded from the statistical analysis because of unrelated underlying medical conditions that produced unreliable data—one severe motor vehicle accident and one pituitary tumor.

**Table 1:** Patient demographics during the study

Parameter	VLF (*n* = 29)	UVF (*n* = 29)
Age (years), mean ± SD	68.90 ± 5.14	67.45 ± 7.07
Age group, *n* (%)		
< 60 years	0.00%	13.79%
60–69 years	51.72%	51.72%
70–79 years	44.83%	34.48%
≥ 80 years	3.45%	0.00%
Sex, *n* (%)		
Male	58.62%	27.59%
Female	41.38%	72.41%
Race, *n* (%)		
Asian	3.45%	6.90%
White	82.76%	86.21%
Other	13.79%	6.90%
Ethnicity, *n* (%)		
Hispanic	13.79%	17.24%
Non-Hispanic	86.21%	82.76%

*n* sample size, *SD* standard deviation, *VLF* violet light filter, *UVF* ultraviolet light filter

### Visual Acuities

**Fig. 1:**
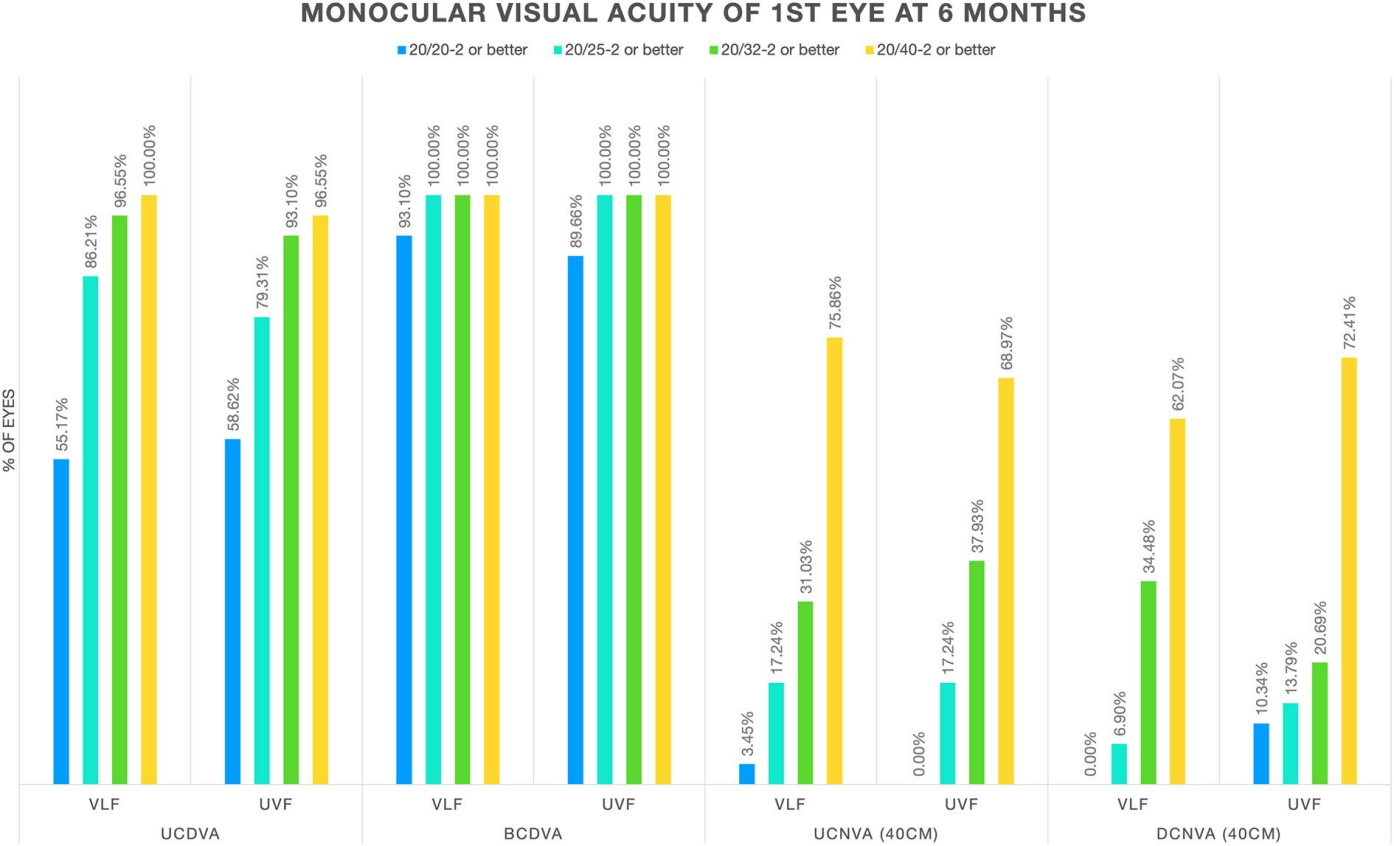
Cumulative monocular uncorrected and corrected visual acuities at far and near distances. *UCDVA *uncorrected distance visual acuity, *BCDVA* best-corrected distance visual acuity, *UCNVA* uncorrected near visual acuity, *DCNVA* distance-corrected near visual acuity, *VLF* violet light filter, *UVF* ultraviolet light filter

Mean distance and near (40 cm) visual acuities for the VLF and ULF groups are shown in Tables 2 and 3, respectively. Cumulative visual acuities for both groups are shown in Fig. 1. No significant differences in visual acuities were noted between the two groups.

**Table 2:** Mean distance visual acuities (logMAR)

Visual acuity	1 month				6 month			
	VLF		UVF		VLF		UVF	
	Mean	SD	Mean	SD	Mean	SD	Mean	SD
Uncorrected monocular^a^	0.04	± 0.09	− 0.05	± 0.14	0.04	± 0.10	0.05	± 0.13
Uncorrected binocular	− 0.01	± 0.09	0.00	± 0.13	− 0.01	± 0.08	− 0.03	± 0.13
Best-corrected monocular^a^	− 0.03	± 0.06	− 0.04	± 0.09	− 0.05	± 0.07	− 0.05	± 0.08

*SD* standard deviation, *VLF* violet light filter, *UVF* ultraviolet light filter

^a^Monocular VA tested using first surgical eye

**Table 3:** Mean near visual acuities (40 cm) (logMAR)

Visual acuity	1 month				6 month			
	VLF		UVF		VLF		UVF	
	Mean	SD	Mean	SD	Mean	SD	Mean	SD
Uncorrected monocular^a^	0.27	± 0.13	0.26	± 0.15	0.28	± 0.12	0.28	± 0.11
Uncorrected binocular	0.16	± 0.12	0.16	± 0.09	0.20	± 0.11	0.17	± 0.10
Distance-corrected monocular^a^	0.31	± 0.12	0.33	± 0.13	0.32	± 0.12	0.30	± 0.14

*SD* standard deviation, *VLF* violet light filter, *UVF* ultraviolet light filter

^a^Monocular visual acuity tested using first surgical eye

**Fig. 2:**
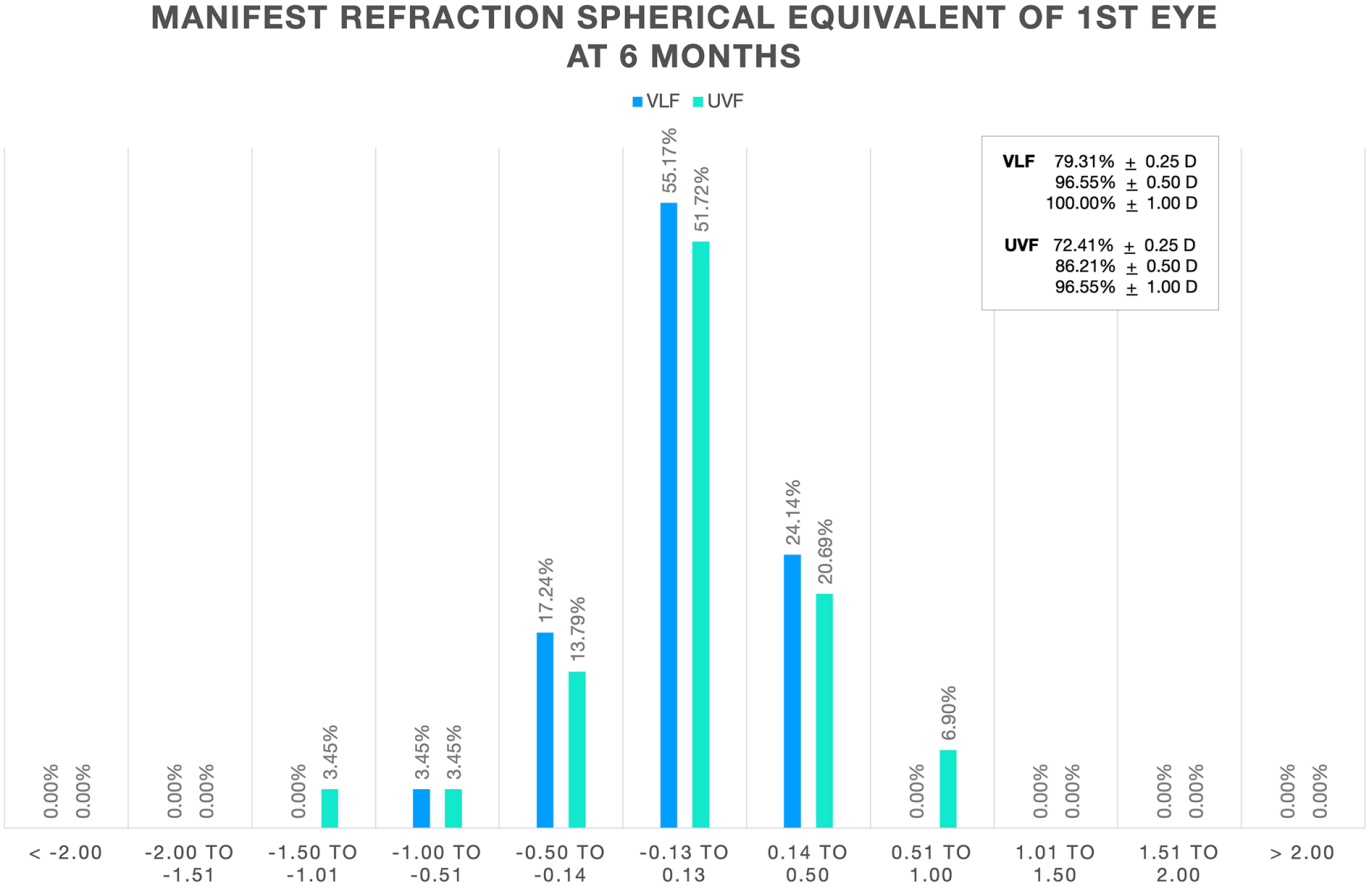
Manifest refraction spherical equivalent distribution. *VLF* violet light filter, *UVF* ultraviolet light filter

**Fig. 3:**
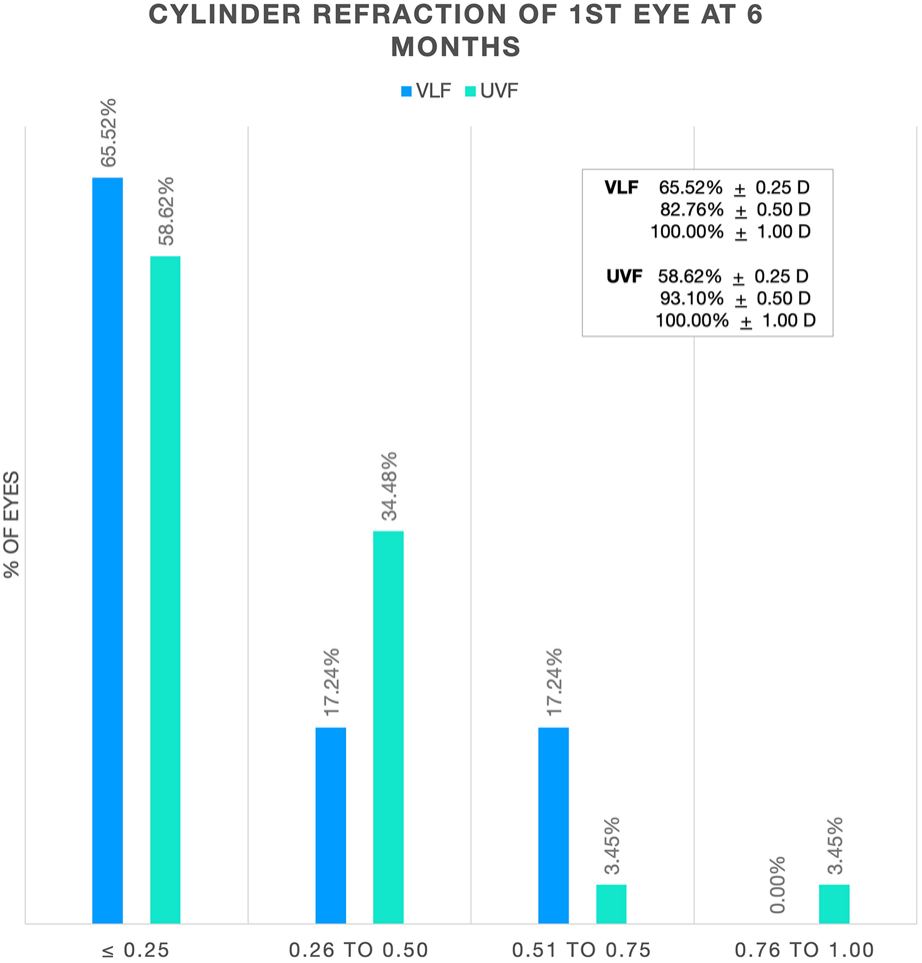
Refractive cylinder distribution at 6 months. *VLF* violet light filter, *UVF* ultraviolet light filter

Manifest refraction spherical equivalent and cylinder refraction at 1 and 6 months are shown in Table 4. At 6 months, the percentage of first eyes with manifest refraction spherical equivalent within ± 0.5 D of emmetropia was 96.55% and 86.21% for the VLF and UVF groups, respectively (Fig. 2). Similarly, the percentage of first eyes with cylinder refractive error ≤ 0.50 D for the VLF and UVF groups was 82.76% and 93.10%, respectively (Fig. 3).

**Table 4:** Manifest refraction spherical equivalent and cylinder correction at 1 month and 6 months (logMAR)

	VLF				UVF			
	1 month		6 month		1 month		6 month	
	Mean	SD	Mean	SD	Mean	SD	Mean	SD
MRSE	− 0.05	± 0.26	0.00	± 0.25	− 0.01	± 0.31	0.02	± 0.38
CYL	− 0.20	± 0.19	− 0.30	± 0.27	− 0.29	± 0.24	− 0.29	± 0.27

*MRSE* manifest refraction spherical equivalent, *CYL* manifest refraction cylinder, *VLF* violet light filter, *UVF* ultraviolet light filter, *SD* standard deviation

### Low Contrast Acuities

Low contrast visual acuities at 6 months are shown in Table 5. No significant differences were noted between the VLF and UVF groups for 10% and 25% low contrast under photopic conditions. Under mesopic conditions, subjects in the VLF group had slightly better 10% low contrast visual acuity, although statistical significance was not reached.

**Table 5:** Low contrast photopic and mesopic visual acuities at 6 months (logMAR)

	VLF		UVF	
	Mean	SD	Mean	SD
Photopic 10%	0.37	± 0.13	0.36	± 0.14
Photopic 25%	0.15	± 0.10	0.15	± 0.18
Mesopic 10%	0.63	± 0.15	0.68	± 0.19
Mesopic 25%	0.40	± 0.12	0.39	± 0.12
Mesopic 25% + glare^a^	1.04	± 0.10	1.03	± 0.14

*SD* standard deviation, *VLF* violet light filter, *UVF* ultraviolet light filter

^a^21/29 Symfony Optiblue, and 22/29 Symfony subjects were unable to see the 20/200 line

During low contrast testing, a small number of subjects were unable to read the largest line of 20/200 (1.0 logMAR). These subjects were imputed a low-contrast acuity of 20/250 (1.1 logMAR) for analysis purposes. Although not significantly different, more of these subjects were noted in the UVF group than the VLF group (Table 5). However, when testing mesopic 25% contrast with glare, 19 UVF and 17 VLF subjects at 1 month, and 22 UVF and 21 VLF subjects at 6 months were unable to read the largest line.

### Visual Symptoms, Spectacle Use, and Patient Satisfaction

Of the patients who reported visual symptoms at 1 month, patients in the VLF group reported significantly less difficulty due to halo (*p* = 0.016) and starburst (*p* = 0.028) symptoms than patients in the UVF group. There were fewer glare complaints in the VLF group at 1 month. By the 6-month visit, dysphotopsia complaints were no longer significantly different between the groups. Of the three patients in the VLF group who reported severe halos and starbursts at 6 months, two reported no halos or starbursts and one reported moderate halos and starbursts at 1 month. The single UVF patient who reported severe halos and starbursts at 6 months had no visual complaints at 1 month (Table 6).

**Table 6:** Spontaneous (non-directed^a^) reports of optical/visual symptoms (first eye) at 6 months

	VLF		UVF	
	*N* = 29		*N* = 29	
	*n*	%	*n*	%
Halos^b^	6	20.69	2	6.90
Mild	2	6.90	0	0.00
Moderate	1	3.45	1	3.45
Severe	3	10.34	1	3.45
Night glare^b^	4	13.79	3	10.34
Mild	1	3.45	0	0.00
Moderate	2	6.90	3	10.34
Severe	1	3.45	0	0.00
Starbursts^b^	7	24.14	5	17.24
Mild	2	6.90	0	0.00
Moderate	2	6.90	4	13.79
Severe	3	10.34	1	3.45
Photophobia	0	0.00	0	0.00
Day glare	2	6.90	0	0.00
Night vision difficulty	1	3.45	1	3.45

% = (*n*/*N*) × 100

*n* sample size for each symptom, *N* overall sample size, *VLF* violet light filter, *UVF* ultraviolet light filter, subjects may report multiple symptoms

^a^Responses to the question, “Are you having any difficulties with your eyes or vision?”

^b^Severity collected in follow-up response

**Fig. 4:**
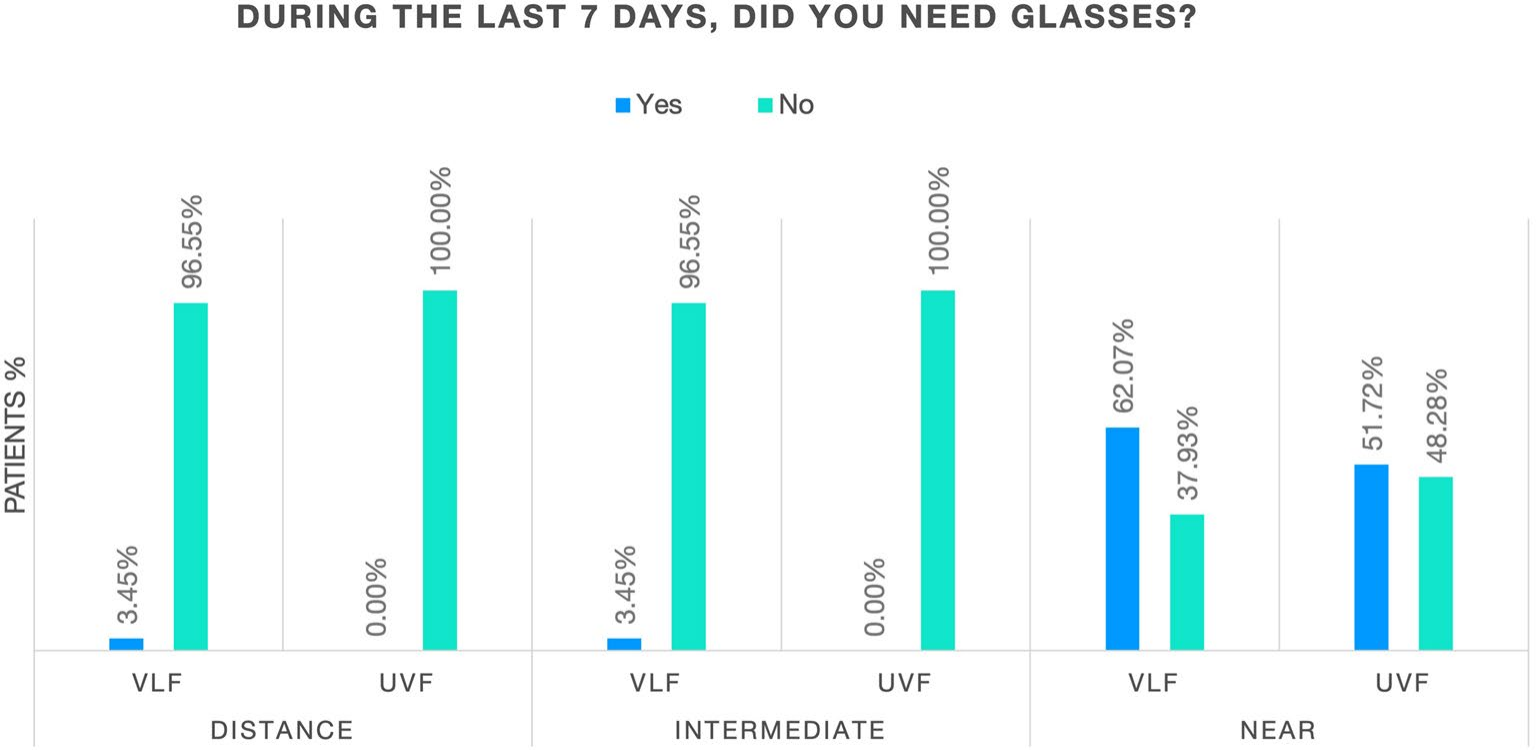
Overall patient-reported spectacle wear at 6 months. *VLF* violet light filter, *UVF* ultraviolet light filter

**Fig. 5:**
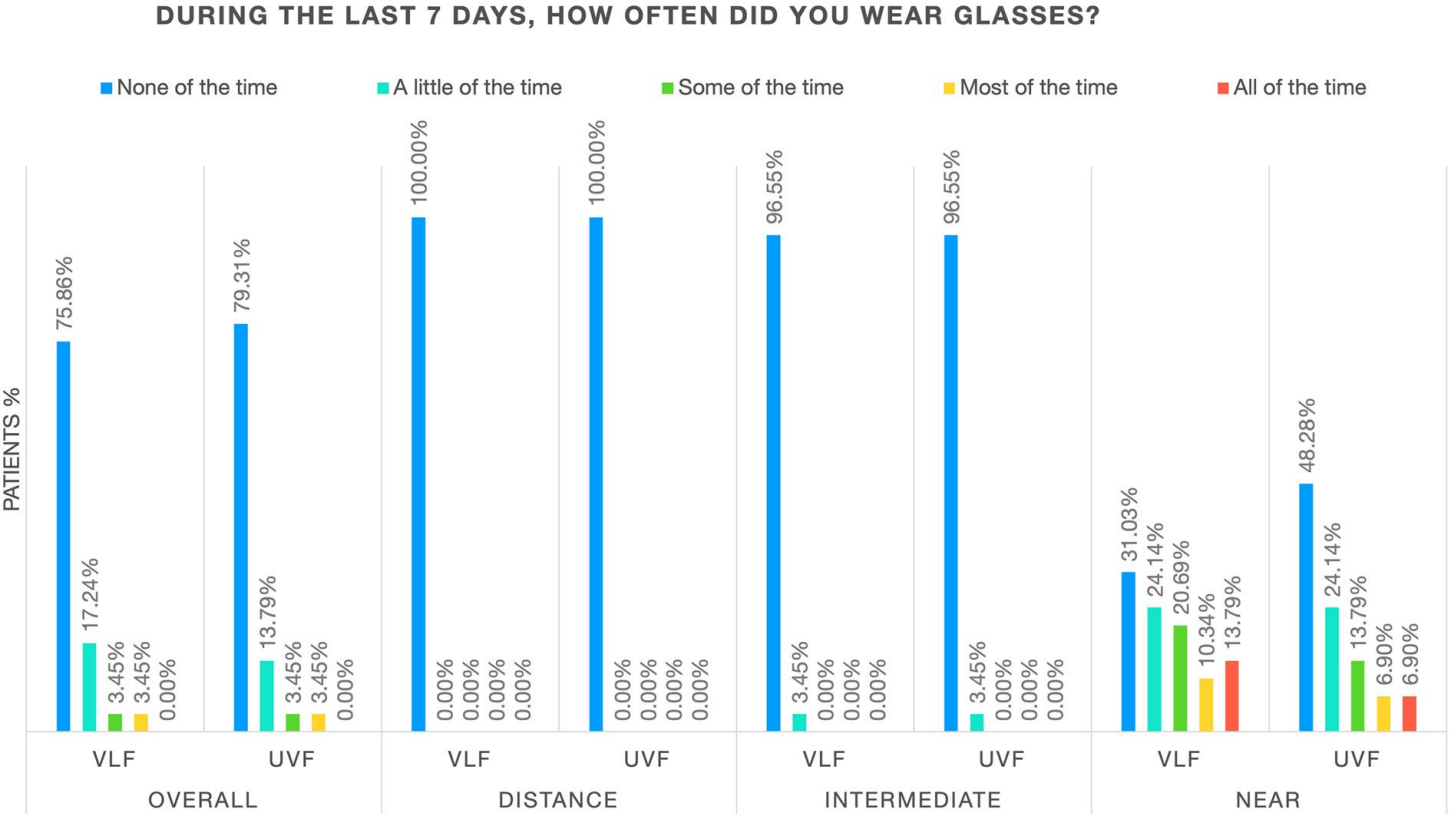
Patient-reported frequency of spectacle wear at 6 months. *VLF* violet light filter, *UVF* ultraviolet light filter

Overall patient-reported spectacle independence and frequency of spectacle wear at 6 months are shown in Figs. 4 and 5. No statistical differences were noted between the VLF and the UVF groups.

Both groups were overall happy with their vision, would recommend to friends/family, and would do this again. At 1 month, the VLF group scored slightly higher than the UVF group, although statistical significance was not achieved (Table 7). Both groups were equally satisfied at 6 months.

**Table 7:** Patient overall satisfaction with intraocular lenses at 1-month and 6-month visits

	1-month visit			6-month visit		
	VLF (%)	UVF (%)	*P* value	VLF (%)	UVF (%)	*P* value
Are you overall happy with your vision?	100.00	93.10	0.15	96.60	96.60	1.00
Would you recommend to your friends/family?	100.00	93.10	0.15	96.60	96.60	1.00
Would you do this again?	100.00	89.70	0.75	96.60	96.60	1.00

*VLF* violet light filter, *UVF* ultraviolet light filter

### Other Endpoints

At 6 months, patients in the VLF required 7.48 ± 2.56 min with the physician and 62.31 ± 10.98 min with the technician. Patients in the UVF required 7.28 ± 2.81 min with the physician and 58.21 ± 7.66 min with the technician. No statistical differences were noted between the two groups.

No secondary procedures (LVC enhancements, YAG capsulotomies, IOL exchanges) were needed in this study.

## Discussion

Presbyopia correction during cataract surgery involves the restoration of functional through-focus [9]. When using pseudo-accommodating lenses, there is a compromise of three interrelated concepts: visual quality, visual range, and visual symptoms (specifically dysphotopsias). The goal is to balance these factors to optimize each patient’s overall visual experience. Previous studies with the Tecnis Symfony IOL (ZXR00) demonstrated high contrast visual acuity and patient satisfaction while providing a broader range of functional vision and greater spectacle independence compared with monofocal IOLs [1, 10, 11]. As a pure EDOF IOL, the Tecnis Symfony has been shown to have improved optical quality, intermediate vision, and fewer dysphotopsias than IOLs with a multifocal component [12].

Dysphotopsias are bothersome low-light photic phenomena most often associated with night driving. The night vision symptoms of glare, halos, and starbursts comprise one of the main causes of patient dissatisfaction with presbyopia-correcting IOLs [11, 13]. Studies reported that while compromises in visual quality and visual symptoms are necessary with presbyopia-correcting IOLs, the severity of these dysphotopsias differ by patient as a result of personality [14] and neuroadaptation [15]. Indeed, while the degree of light scatter and resulting retinal veiling luminance is a function of the IOL [8], the perception and severity of dysphotopsia symptoms are mediated at the level of the visual cortex [7].

An additional challenge to understanding dysphotopsias is the subjective nature of the testing process. While there are objective testing methods [16], most studies on dysphotopsias are based on the subjective patient-reported outcomes (PRO) data [2, 3, 14, 17, 18]. Obtaining accurate and consistent responses from patients recalling their night vision symptoms in a research setting can be a challenge, often resulting in mischaracterizations and internally inconsistent responses. For example, although non-directed reports of halos and starbursts were consistent between the UVF group and the previously reported rates [2], they were much higher in the VLF group. However, two of the three patients in the VLF group who reported severe halos and starbursts at 6 months reported “mild” or “none” at 1 month. Patients often demonstrate confusion with the written descriptions of glare, halos, and starbursts. Newer instruments are being developed, some of which include photographic representations to improve accuracy and consistency [19–21].

High-energy violet light is more susceptible to scatter than lower-energy wavelengths. Therefore, violet light filtration can help to mitigate dysphotopsia symptoms [4–6]. In a benchtop study, the VLF ZXR00V showed a 19% improvement in halo performance and a 12–17% reduction in retinal veiling luminance from stray light versus UVF ZXR00 [8]. The current study showed a notable improvement in dysphotopsia symptoms at the 1-month visit in the VLF versus the UVF group. Although this difference was no longer statistically significant at 6 months, the reduction in light scatter in the VLF group may account for the earlier neuroadaptation with the VLF group. Objective clinical testing of photic phenomena and their relationship to dysphotopsia complaints may help further our understanding of these symptoms.

With regards to low contrast visual acuity, benchtop further demonstrated a 13–19% improvement in contrast vision with the VLF ZXR00V versus the UVF ZXR00, particularly under challenging conditions [8]. While clinical significance was not reached in the current study, mesopic 10% low contrast visual acuity favored the VLF group at 1 month. Data from low contrast visual acuity with glare testing was not usable because over two-thirds of subjects in both groups were unable to see the largest optotype at the only glare setting available on the CTS system. Improvements in low contrast testing equipment and methodology with lower and perhaps adjustable brightness and color temperature glare testing would be beneficial to understand dysphotopsia under more real-world conditions.

Seasonal variations in daylight hours may impact dysphotopsia and patient satisfaction reporting as a result of varied needs for driving in low-light conditions. Clinical studies on dysphotopsias are conducted throughout the year, and when patients spend more time driving in low-light conditions in the fall and winter seasons, they may be apt to report more dysphotopsia symptoms and dissatisfaction with night vision. While the statistical data showed no significant group effect in the present study, there may yet be a seasonal effect; three of the four subjects who were extremely bothered by dysphotopsias in this study demonstrated seasonal variability consistent with this hypothesis.

The VLF group reported 100% satisfaction at the 1-month visit thus not requiring any additional unscheduled postoperative visits. The UVF group had 89.70% satisfaction at the 1-month visit. In this case, the patients who did not report satisfaction required at least one additional visit, and in some cases multiple visits, to achieve satisfaction. Earlier patient satisfaction would likely reduce overall clinic chair time for surgeons and could even positively impact overall practice financials, as reported by Chang et al. in “Clinical Performance of a Diffractive Extended Depth of Focus IOL with a Violet Light Filter and High-Resolution Lathing,” a poster presented at the ASCRS annual meeting in May 2023.

### Limitations

There are several limitations in the study. The small sample size limits the power and has a higher potential for outliers to affect the reliability of results, making some results appear more prevalent than they would in a larger population. For example, 100% satisfaction would not likely be the case in larger studies. Additionally, inconsistencies in PRO responses limit the investigators’ ability to interpret study results and inform patient care in the future.

Furthermore, glare-testing was only conducted using a single brightness and color temperature. Glare sources with other emission spectra may highlight potential differences with variations in chromophores. Improvements in testing modalities of low contrast visual acuity, especially with glare, as well as objective testing of dysphotopsia symptoms may be warranted.

## Conclusion

Overall visual outcomes and patient satisfaction were excellent in both groups. Violet light filtration and manufacturing changes demonstrated small but significant improvements in the clinical performance of the Tecnis Symfony Optiblue IOL over the Tecnis Symfony IOL. In particular, the Tecnis Symfony Optiblue IOL reduces light scatter and thus dysphotopsia symptoms earlier in the postoperative period. Although clinical significance was not reached, the trend towards clinical benefit in low contrast acuity warrants further investigation with modalities that offer additional contrast and glare testing levels.

## Data Availability

The datasets generated during and/or analyzed during the current study are available from the corresponding author on reasonable request.
